# From cloning to mutant in 5 days: rapid allelic exchange in *Staphylococcus aureus*

**DOI:** 10.1099/acmi.0.000193

**Published:** 2021-01-07

**Authors:** Ian R. Monk, Timothy P. Stinear

**Affiliations:** ^1^​ Department of Microbiology and Immunology, The University of Melbourne at the Peter Doherty Institute for Infection & Immunity, Melbourne, Victoria, Australia

**Keywords:** allelic exchange, Electroporation, mutagenesis, Staphylococcus aureus, temperature sensitive, Transformation

## Abstract

In the last 10 years, the barriers preventing the uptake of foreign DNA by clinical *Staphylococcus aureus* isolates have been identified and powerful mutagenesis techniques such as allelic exchange are now possible in most genotypes. However, these targeted approaches can still be cumbersome, and the construction of unmarked deletions/point mutations may take many weeks or months. Here, we introduce a streamlined allelic exchange protocol using IMxxB *Escherichia coli* and the plasmid pIMAY-Z. With this optimized approach, a site-specific mutation can be introduced into *S. aureus* in 5 days, from the start of cloning to isolation of genomic DNA for confirmatory whole-genome sequencing. This streamlined protocol considerably reduces the time required to introduce a specific, unmarked mutation in *S. aureus* and should dramatically improve the scalability of gene-function studies.

## Introduction

The introduction of unmarked, site-directed mutations *in vivo* through double crossover allelic exchange (AE) is a powerful tool used by molecular microbiologists working with staphylococci. Mutations in the area of interest, flanked by regions of homology to the genome under investigation, are constructed in a temperature-sensitive plasmid. The plasmid is introduced into *S. aureus* by electroporation, and single crossover integration is selected at a temperature non-permissive for plasmid replication. Subsequent plasmid excision is stimulated by growth at a temperature that permits rolling circle replication of the plasmid, resulting in either a wild-type or mutant phenotype [1].

In 2012, we described the construction of *E. coli* strain DH10BΔ*dcm* (called DC10B) [2]. The deletion of the cytosine methylase from the high-efficiency cloning strain allowed direct cloning and subsequent transformation of the majority of *S. aureus* strains tested, bypassing the strong type IV restriction barrier [2]. However, passaging plasmids through DC10B does not evade the heterogeneous type I systems present in all *S. aureus* strains. Additionally, we constructed a novel AE plasmid pIMAY, containing the following features: (i) pWV01 temperature-sensitive replicon from *Lactococcus lactis* (cannot grow at 37 °C in Gram-positive hosts), (ii) low copy p15a *E. coli* replicon, (iii) constitutively expressed *cat* resistance marker and (iv) anhydrotetracycline (ATc) inducible antisense *secY* for counter selection. The application of DC10B and pIMAY streamlined the construction of mutants in *S. aureus* [3–5], *Staphylococcus epidermidis* [6], *Staphylococcus lugdunensis* [7]. Through expression of the *hsdMS* alleles responsible for lineage-specific adenine-methylation profiles, we further improved the DC10B host to create the IMxxB strains. These strains mimic the clonal complex (CC)1 (IM01B), CC8 (IM08B), CC30 (IM30B) or ST93 (IM93B) adenine methylation, permitting high-efficiency transfer of plasmid into *S. aureus* strains containing the same complement of *hsdMS* genes. The incorporation of *lacZ* into pIMAY (yielding pIMAY-Z) enabled phenotypic selection for plasmid loss, simplifying screening, and removing the need for ATc induction of the antisense *secY*. The combination of IMxxB strains and pIMAY-Z has improved plasmid transfer and simplified the steps for the construction of targeted mutations [8].

Important factors to consider when employing AE are as follows: (i) temperature sensitivity of the Gram-positive replicon; (ii) level of antibiotic selection required to select as a single copy; and (iii) a means for plasmid loss to be identified or selected. Additional factors to consider include the following: the presence of endogenous restriction barriers present within different *S. aureus* clonal complexes, that require bypass for introduction of plasmid; and efficiency of the total process. Here, we detail an optimized approach, for the rapid construction of deletions, point mutations and genome insertions into the chromosome or plasmids of *S. aureus* in 5 days.

## Methods

### Media and culture conditions

Bacteria were routinely grown at 37 °C with shaking (200 r.p.m.) unless overwise stated. IMxxB *E. coli* strains have been deposited and are available from BEI Resources (https://www.beiresources.org/) and the Belgium Co-ordinated Collections of Microorganisms (http://bccm.belspo.be/). The plasmid pIMAY-Z is also available at the Belgium Co-ordinated Collections of Microorganisms. [NOTE 1. The primary advantage of pIMAY-Z is the thermosensitivity of the plasmid, however, others have noted that mutations can arise in the Gram-positive replicon, which lead to inactivation [9]. These mutations are linked with growth of the plasmid in *E. coli* at temperatures lower than 37 °C due to the presence of two active replicons. We recommend only growing pIMAY-Z transformed *E. coli* at 37 °C and not leaving broth or plate cultures on the bench].

### Rubidium chloride competent *E. coli *.

The method for the transformation of *E. coli* is based on the protocol of Green and Rodgers [10], which is derived from Hanahan [11]. An overnight 10 ml L-broth (LB 30 ml universal) of an IMxxB [8] strain was grown at 37 °C. A 500 ml culture of prewarmed LB (2 l conical flask) was inoculated with 5 ml of the overnight and grown at 37 °C to an OD_600_ of 0.5 (about 3–4 h). The culture was centrifuged at 7000 ***g***, 4 °C for 5 min with 30 ml of the supernatant was used to combine the pellets. After 15 min on ice, the cells were centrifuged at 7000 ***g***, 4 °C for 5 min and the supernatant discarded. A 30 ml aliquot of ice cold TfbI (100 mM RbCl, 50 mM MnCl_2_-4H_2_O, 30 mM CH_3_CO_2_K, 10 mM CaCl_2_-2H_2_O, 15% w/v glycerol, filter sterilized 0.2 µM) was used to resuspend the cell pellet and left on ice for 15 min. The cells were centrifuged at 7000 ***g***, 4 °C for 5 min and the supernatant discard. The cells were resuspended in 8 ml of ice cold TfbII [10 mM MOPS pH 6.5 (0.2 M filter sterilized stock), 10 mM RbCl, 75 mM CaCl_2_-2H_2_O, 15 % w/v glycerol, filter sterilized 0.2 µM], aliquoted (80 µl) and frozen at −70 °C.

### SLiCE extract isolation

The SLiCE (Seamless Ligation Cloning Extract) [12] was isolated from *E. coli* strain DY380 [13] grown in 50 ml 2xYT (1.6% Tryptone, 1% Yeast Extract, 0.5% NaCl) at 30 °C after a 1:100 dilution of the overnight 30 °C 2xYT culture. Once the culture reached an OD_600_ of ∼2.5 (under our culture conditions the saturated culture would reach an OD_600_ of ~4), the cells were moved to 42 °C for 25 min (with the addition of 50 ml of 2xYT prewarmed to 42 °C). The cells were centrifuged (7000 ***g*** for 10 min at 4 °C) and washed twice with an equal volume of ice-cold mQH_2_O. The pellet was lysed in 500 µl of CellLytic B cell lysis reagent (C87040; 10 ml; Sigma) and incubated at room temperature for 10 min on a rotating platform. Cellular debris was pelleted (17 000 ***g*** for 1 min) and the supernatant added to an equal volume of 100% sterile glycerol. Working aliquots of the SLiCE extract (100 µl) were stored at −20 °C, while long-term aliquots were stored at −70 °C. No reduction in SLiCE activity was observed, even after 6 months at −20 °C. [NOTE 2. We routinely use *E. coli* strain DY380 for the isolation of SLiCE as described above, however, it is possible to use other *E. coli* strains (PPY, DH10B+pKD46), which possess the lamba red functions [12, 14]. Extracts from other *recA-*cloning strains of *E. coli* have been reported to permit cloning [15]. Purification of the SLiCE treated cloned DNA prior to transformation has been shown to improve transformation efficiency [16]].

### Amplification of pIMAY-Z backbone

To clone into pIMAY-Z [8], the plasmid was first linearized with KpnI (37 °C for 4 h) and the 8.8 kb band gel extracted (Monarch gel extraction kit, NEB). The vector backbone (5 ng µl^−1^) was amplified from within the multiple cloning site using primers IM1/IM2 (Table 1) with 0.5 U of Phusion (NEB) and 0.25 U of Phire hotstart II DNA polymerase (Thermo Fisher) with annealing at 50 °C and extension for 3 min for 30 cycles. A large PCR (250 µl) was conducted, then 1 µl of DpnI was added directly to the PCR reaction and incubated 1 h at 37 °C. The amplimer was PCR purified (Monarch PCR and DNA Cleanup Kit column, NEB) and eluted in 100 µl and then stored at −20 °C (50–100 ng µl^−1^). The vector was functional for SLiCE cloning for over 2 years. Using this protocol, we routinely observed no background (vector without insert) in SLiCE cloning. [NOTE 3. It is essential that the plasmid is digested to completion with KpnI to prevent plasmid carryover. To amplify pIMAY-Z, we combine the editing function of Phusion and the processivity of Phire. Without Phire we were unable to consistently amplify the backbone. Minor additional bands are observed at 3 kb and below, with the major band at 8.8 kb (Fig. S2, available in the online version of this article)].

**Table 1.** Primers

	5′−3′ sequence
IM1	GGTACCCAGCTTTTGTTCCCTTTAGTGAGG
IM2	GAGCTCCAATTCGCCCTATAGTGAGTCG
IM3	AATACCTGTGACGGAAGATCACTTCG
IM4	TACATGTCAAGAATAAACTGCCAAAGC

### Amplification of insert by splicing by overlap extension (SOE)-PCR or gBlock

To delete a region of DNA, it was first examined for gene context. In most cases, where overlap was not observed with the preceding or proceeding gene, the entire ORF was deleted leaving the start and stop codon. Where overlap was observed, the deletion was moved in frame leaving five codons at the 5′ and or 3′ end. A minimum of 500 bp was required to flank either side of the mutation. For the insertion of point mutations into essential genes, the entire essential ORF was cloned with the 500 bp rule observed. We have also used synthesized gBlocks (IDT) encompassing the modification, which can further speed up the process, with these are reconstituted at 100 ng µl^−1^ and used directly. For point mutations, additional neutral changes were introduced (where possible and required) to facilitate a PCR screening approach. The complementary mutation was present in the 3′ end of the forward screening primer, with this a perfect match to the mutation leading to a difference in annealing temperature and used in combination with the ‘D’ reverse primer (see below). The difference annealing temperature could be determined on wild-type chromosomal DNA versus mutated region cloned into pIMAY-Z with a gradient PCR. Point mutation screening conditions were established with a gradient PCR (e.g. annealing at 50, 55, 60, 65, 70 °C) on wild-type genomic DNA or the pIMAY-Z(insert) mutated template. A temperature which amplified from the pIMAY-Z construct, but not genomic DNA, was used in the colony PCR screen. [NOTE 4. For example, when we introduce of mutations into the essential histidine kinase *walK* (gene size 1.8 kb), if the mutation is more than 500 bp into the gene or before the end we would clone from the start codon down to the stop codon. However, if it is less than 500 bp from the start, as the upstream gene is also essential (response regulator *walR*), we would include the entire upstream ORF from the start codon also. Gene essentiality can be inferred from TraDIS or TN-seq studies [17, 18]].

A ~500 bp sequence upstream (or the entire essential ORF) of the gene to be deleted or point mutation was amplified with oligonucleotides A and B (A/B) (up to the start codon or point mutation) and the downstream sequence with oligonucleotides C/D (down from the stop codon or point mutation) separately. The A (5′-CCTCACTAAAGGGAACAAAAGCTGGGTACC+gene specific primer) /D (5′-CGACTCACTATAGGGCGAATTGGAGCTC+gene specific primer) primers are tailed with sequence complementary to the IM1/IM2 primers used to amplify pIMAY-Z. The upstream and downstream PCR products were diluted 1:20 together in water (for point mutations, the AB and CD product were gel extracted), and 1 µl used as the template in a second SOE PCR with the A/D primers. The AD product was gel extracted and 1 µl of each (~10 ng) used in a SLiCE reaction. The AB/CD products can be used in a SLiCE reaction directly but transform at a reduced efficiency (a 50–90 % reduction).

### SLiCE cloning

In a 10 µl reaction, the following components were added, 1 µl 10× ligation buffer (NEB), 1 µl pIMAY-Z (50–100 ng µl^−1^), 1 µl of gel extracted insert (10–100 ng µl^−1^) or gBlock (100 ng µl^−1^), 6 µl of mQH_2_O and 1 µl of SLiCE and mixed by pipetting. The first time the PCR amplified pIMAY-Z was used, the insert is omitted to verify the level of background. The SLiCE reaction was incubated at 37 °C for 15 min and then stored on ice. SLiCE reactions can be stored at −20 °C prior to transformation.

### Transformation of *E. coli*

To transform, an aliquot of competent *E. coli* was thawed on ice and 10 µl of the SLiCE reaction was added. The cells were incubated on ice for 30 min. The suspension was mixed by gently flicking every 10 min. A heat shock at 42 °C for 45 s was preformed and then placed on ice for 2 min. To the cells, 500 µl of LB was added and incubated (37 °C, 200 r.p.m.) for 1 h. If directly selecting the transformants in liquid, 25 ml LB (chloramphenicol 10 µg ml^−1^) was inoculated with 50 µl of regenerated cells and incubated overnight at 37 °C. A 100 µl aliquot was also spread plated onto Brain Heart Infusion agar (Difco) containing 10 µg ml^−1^ chloramphenicol and X-gal (Bioline) 100 µg ml^−1^ (BHIA-CX) and incubated at 37 °C overnight. Two blue colonies were single colony purified (SCP) on BHIA-CX and then screened by colony PCR with IM3/IM4 (Table 1). [NOTE 5. The IM3/IM4 PCR will add an additional 190 bp to the size of the insert].

### Plasmid DNA purification

Plasmid was isolated from 25 ml of culture with the Monarch plasmid DNA miniprep kit (NEB) using 2.5 times the volume of buffers B1, B2 and B3, with the lysate put through one column and processed as described by the manufacturer. The DNA was eluted in 30 µl of prewarmed (60 °C) elution buffer (10 mM Tris.Cl pH 8.5) and the DNA quantified with the BR dsDNA Qubit kit (Invitrogen). If an insufficient concentration of plasmid DNA was obtained (less than 500 ng in 10 µl), the plasmid was concentrated over a Monarch PCR and DNA Cleanup Kit column (NEB) and eluted in 6 µl in prewarmed elution buffer.

### Electrocompetent *S. aureus*

Overnight cultures of *S. aureus* were grown in 10 ml of Brain Heart Infusion broth (BHI-Oxiod) in a 50 ml tube (37 °C, 200 r.p.m.) and then diluted to an optical density at 600 nm of 0.5 (Biophotometer, Eppendorf) in fresh prewarmed media. The cultures were incubated for a further 40 min. All subsequent steps were performed at 4 °C. The cells were harvested at 7000 ***g*** for 5 min, supernatant discarded, and an equal volume of autoclaved ice-cold water added (repeated twice). The cells were centrifuged and resuspended in 1/5 vol of autoclaved ice-cold 10% (w/v) glycerol (repeated twice) and finally in 1/200 volume. Aliquots of 50 µl were frozen at −70 °C. [NOTE 6. For some strains finally concentrating 1:400 rather than 1:200 can improve the transformation efficiency. However, concentrating cells may lead to arcing with some strains and requires empiric testing].

For electroporation, cells were thawed on ice for 5 min, centrifuged (5000 ***g*** for 3 min) and resuspended in 80 µl of 10% glycerol and 500 mM sucrose (filter sterilized). Plasmid DNA (0.5–1 µg in up to 10 µl) was added to the cells, transferred to a 1 mm electroporation cuvette (Bio-Rad) at room temperature, and pulsed at 21 kV/cm, 100 ohms, and 25 µF (BTX 630, BTX or Xcell, GenePulsar I/II, Biorad). To the cells, 1 ml of BHI supplemented with 500 mM sucrose (BHIS - filter sterilized) was added immediately to the cuvette (pipetted up and down three times), transferred to a 15 ml tube and incubated tilted (45°) at 30 °C for 1 h (200 r.p.m.) before plating two 100 µl aliquots on BHIA+CX (Chloramphenicol 10 µg / Xgal 100 µg ml^−1^). These plates were either incubated at 30 °C for 48 h or 37 °C overnight. The remaining cells were concentrated (7000***g*** for 5 min), resuspended in 100 µl of BHIS and plated on BHIA+CX and incubated overnight at 37 °C.[NOTE 7. In general, the addition of more ‘clean’ plasmid will yield more transformants up to a threshold of 5 to 10 µg [8]. With plasmid isolated from IM08B into a CC8 strain of *S. aureus *, we routinely observe 10^4^ to 10^5^ c.f.u. µg^−1^, which will yield direct integration of pIMAY-Z].

## Allelic exchange

### Fast (2020) integration

Direct integration of pIMAY-Z was selected after transformation and plating on BHIA-CX at 37 °C. On these plates, large blue colonies (direct integrants) arose in a background lawn of small white colonies. A total of six blue colonies were SCP onto BHIA-CX and incubated at 37 °C. Of the six, two well-isolated colonies from the integration plate were also inoculated into 10 ml BHI broth and incubated at 30 °C (200 r.p.m.) to simulate plasmid excision.

### Slow (2015) integration

For strains that do not permit direct integration, a colony from the 30 °C plate (after 48 h), was homogenized in 200 µl of PBS and the suspension diluted tenfold to 10^−4^. A 50 µl aliquot of the homogenized colony was spread plated on half a BHIA-CX and 10 ul spots from the dilutions were run down on the second half of the plate and incubated overnight at 37 °C (integration plate). A 30 °C master plate was also made by SCP on BHIA-CX. A total of six blue colonies were SCP on BHIA-CX at 37 °C from the integration plate. The presence of the correct sized insert (colony PCR with IM3/IM4) on the 30 °C master plate and the side of plasmid integration on the 37 °C SCP plate were determined for deletion constructs with primers IM3/D reverse or IM4/A forward primers. This is useful as one side of integration usually yields a mutant at a higher efficiency than the other. We normally go through the process and come back to identify the screen side of integration if a mutant is not obtained from round one of screening. [NOTE 8. Colonies that arise on the 30 °C transformation plate are often a mixture of white and blue. Upon integration at 37 °C, white colonies will normally turn blue].

A clone with either side of integration (if possible) from 37 °C SCP plates were inoculated into 10 ml BHI broth and grown at 30 °C to simulate plasmid excision.

### Plasmid excision

The 30 °C broths were grown to saturation (~8 h or overnight) and diluted with 50 µl of the 2×10^−5^ and 1×10^−6^ dilutions spread plated onto BHIA containing X-gal (BHIA-X) at 100 µg ml^−1^ and incubated overnight at 37 °C. Under these conditions, plasmid excision can vary, but normally 5–10 (or more) isolated white colonies can be distinguished . White colonies were screened by colony PCR with the AD primers (for gene deletion and insertion) or point mutation screening primers (the forward primer containing the 3′ match to the mutation and the D primer), SCP onto BHIA-X agar plates and incubated at 37 °C overnight. [NOTE 9. The level of plasmid excision can vary dramatically between constructs and even clones of different integrants. The number of white colonies obtained does not always correlate with the number of mutants isolated. Where integration has a fitness cost, excision of the plasmid is normally improved].

### Colony PCR on *S. aureus*

A very small amount of a colony was transferred to the side of a PCR tube, 50 µl of the PCR master mix added (0.5 U Phire Hotstart II Green buffer, Thermo fisher) and a 35 cycle PCR conducted. In the master mix, 200 nM of each primer was used. The only change to the manufacturer’s instructions was extending the initial 98 °C denaturation from 30 s to 3 min.

### Isolation of genomic DNA

A 10 ml BHI culture was inoculated with a colony from a presumptive mutant and grown to saturation at 37 °C (~4 h). From the culture, 1 ml was centrifuged (7000 ***g*** for 2 min), resuspended with 1 ml of PBS and centrifuged. The supernatant was discarded and the cell pellet was resuspended in 100 µl of PBS (containing 100 µg ml^−1^ of RNase A, 10 µg ml^−1^ lysostaphin) and incubated 10 min at 37 °C. The lysate was then processed through the Monarch genomic DNA purification kit (NEB), following manufacturers’ instructions. The PCR for the mutation was repeated and the DNA used forwhole genome sequencing (WGS). The remaining culture was stored at −70 °C in 15% glycerol.

## Results and discussion

**Fig. 1.**
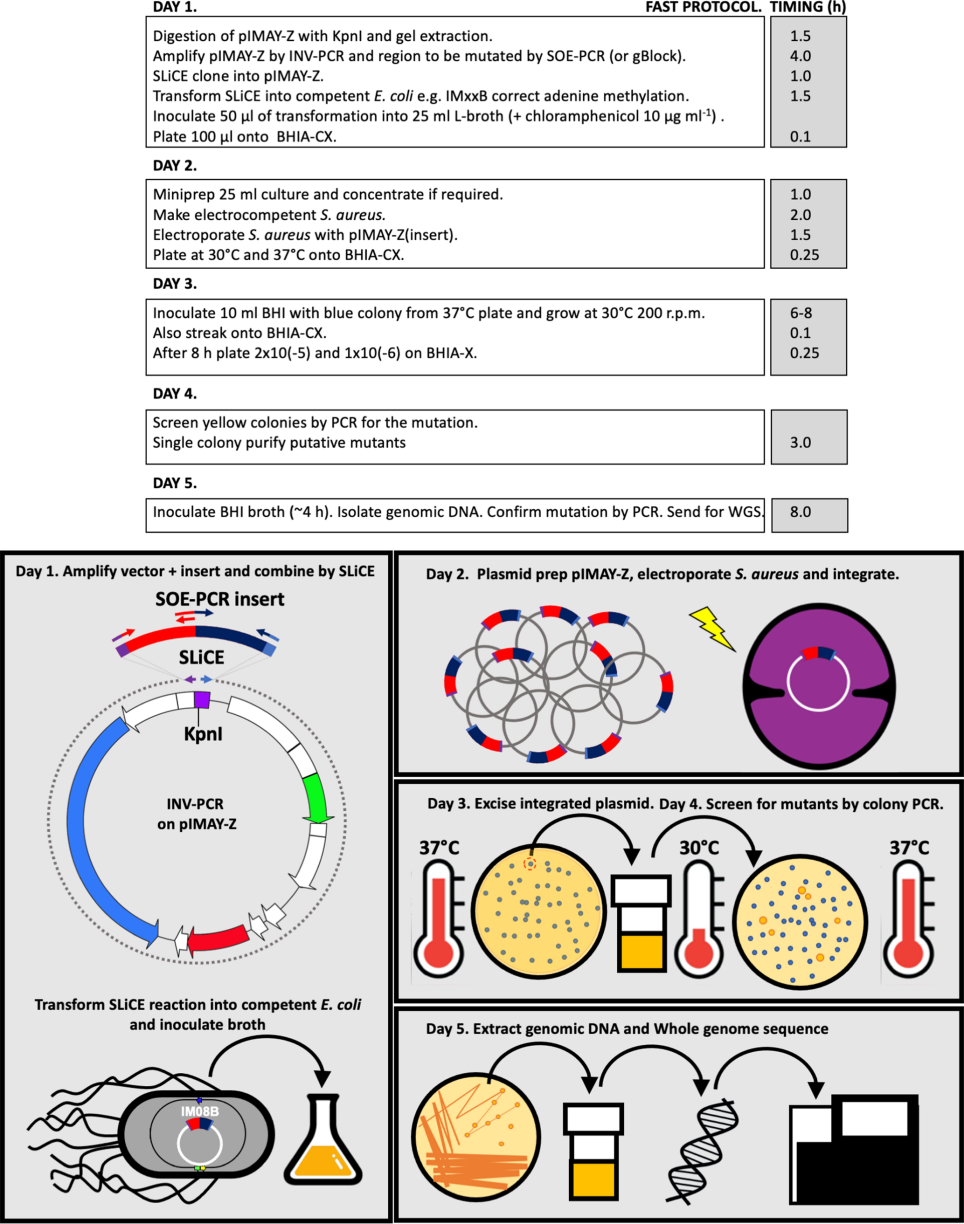
Flow chart for the *S. aureus* 5 day allelic exchange protocol.

Utilization of AE plasmid pIMAY-Z and IMxxB strains of *E. coli* to bypass restriction modification in different *S. aureus* clonal complexes have previously been described [8]. Through further improvements and refinements to the protocol for cloning into pIMAY-Z, direct selection of chromosomal integration after electroporation and condensing the steps for plasmid excision, we have reduced the time to isolate a mutant from 13 down to 5 days (Figs 1 and S1).

The first step in streamlining the protocol was the application of Seamless Ligation Cloning Extract (SLiCE) to expedite preparation of the mutagenesis construct [12]. The benefits of SLiCE are as follows. (i) Cost – SLiCE can be made cheaply and yields a reproducible method to introduce DNA into a vector. (ii) Reproducibility – a pIMAY-Z stock can be PCR amplified in large quantities and is extremely stable, which means the same template can be used repeatedly. We have used the same batch of freeze-thawed vector for over 2 years without a reduction in cloning efficiency. (iii) Speed – the recombination reaction is fast and requires only one 15 min incubation and can be directly transformed into *E. coli* without purification. (iv) Efficiency – with appropriately amplified vector we observe zero background. This protocol allows for the direct plasmid isolation of the methylated AE construct (in the appropriate IMxxB strain) after dilution of the transformed regenerated cells in fresh media with antibiotic selection. We have recently described a compendium of methylation profiles for *S. aureus* that can assist selection of the best IMxxB strain [6]. If no appropriate *E. coli* strain is available with at least one of the endogenous methylation profiles for the *S. aureus* isolate to be manipulated, we routinely use IM08B for cloning. Equivalent to *S. aureus* strain RN4220, IM08B is chosen as it enables high-efficiency plasmid transfer into commonly used *S. aureus* strains (e.g. HG003, Newman, LAC, NRS384), with a transfer efficiency comparable to *E. coli* strain DC10B into other clonal complexes.

Direct selection for chromosomally integrated plasmid on plates at 37 °C, post-electroporation, was an additional refinement; bypassing the isolation of *S. aureus* cells containing replicating plasmid at 30 °C, a step that typically adds 2 to 4 days. We routinely use direct selection in strains with high-efficiency electroporation, such as Newman and NRS384. However, we have found direct selection is not possible in strains with an electroporation efficiency below 10^4^ c.f.u. µg^−1^ of plasmid (e.g. MW2, or VISA mutants of NRS384-thickened cell wall with pRAB11 [19] or similar). For these strains we suggest optimising the electroporation or constructing a *hsdR* mutant [8] to improve plasmid transfer into the target strain. The ability to select for integration at 37 °C (rather than 42 °C and above [20–23]) reduces the potential for the isolation of off-target secondary mutations [24]. Such mutations are also minimised by the level of antibiotic selection afforded by pIMAY-Z as a single copy in the chromosome [2, 8]. Direct plasmid integration may also be improved by increasing the length of sequence homology in the cloned arms, e.g. 1 kb vs 0.5 kb, however this was not empirically tested.

We determined pIMAY-Z as superior to our previously described allelic exchange vector, pIMAY [25]. The pIMAY construct requires ATc induction of antisense *secY* for excision, without ATc induction the integration of pIMAY in the chromosome is very stable. Issues with unreliable plasmid excision when using antisense counterselection with *secY* were recently highlighted [13]. In contrast to pIMAY, the excision and plasmid lost observed with pIMAY-Z was consistent with reasonable efficiency, although subject to some construct variability. The addition of constitutive LacZ for phenotypic selection in pIMAY-Z in combination with uninduced leaky *secY* is enough of a burden to the cell to help stimulate plasmid excision. The ability to induce excision after one passage in broth culture at 30 °C and plating at 37 °C further accelerates the mutant construction process. Besides the time saved, removal of ATc induction reduces cost and decreases the chance of second site mutations due to prolonged non-selective growth. After colony PCR screening and then confirmation of the result with a second PCR on genomic DNA, we would normally perform WGS on two independent mutants (from different 30 °C excision cultures). The WGS step confirms the correct change has been introduced and that there are no off-target mutations.

There are numerous approaches to modify the genome of *S. aureus *, with traditional AE [21, 26–28], the advent of CRISPR [29–35] and other novel applications [36–39]. In the most recently described protocol for allelic exchange with a temperature sensitive plasmid from Austin and Bose [40] 9 days is required post-target strain transformation. This is a similar time frame to the slow protocol; however, a higher temperature is needed for plasmid integration and sequential rounds of broth passage is required to excise the plasmid. The application of CRISPR yields a rapid positive selection within *S. aureus *. The current approach for gene deletion requires two rounds of cloning in *E. coli* for the guide RNA and homology arms. Additionally, as described in the protocol, mutagenesis requires the passage of plasmid through RN4220 prior to plasmid isolation and then electroporation or transduction into the target strain. A new approach for base editing has been developed for the introduction of stop codons into a gene without the requirement for homology arms, but the flexibility of this limited to the due to the requirement of a NGG motif 13–17 bases downstream from the site to be edited [41].

We have found that the combination of IMxxB *E. coli* and pIMAY-Z yield powerful tools for the rapid flexible targeted mutagenesis of *S. aureus* without the limitations of other approaches, such as the necessity for a proximal PAM motif for CRISPR [31, 32]. Additionally, we have found that the 2015 protocol (Fig. S2) is directly applicable to Enterococci [42, 43] and *Listeria monocytogenes* (data not shown), with the potential for further optimization.

## Supplementary Data

Supplementary material 1
