# Supplementary material for: From cloning to mutant in 5 days: rapid allelic exchange in Staphylococcus aureus
Source: Access Microbiol. 2021 Jan 7;3(2):000193. doi: 10.1099/acmi.0.000193 (PMC8209637; doi:10.1099/acmi.0.000193)

Supplementary Figure 1. Protocol from Monk et al 2015

DAY 1.
Digestion of pIMAY-Z with KpnI and gel extraction.
Amplify pIMAY-Z by INV-PCR and region to be mutated by SOE-PCR (or gBlock).
SLiCE clone insert into pIMAY-Z.
Transform into competent *E. coli* eg. IMxxB for correct adenine methylation.
Plate 100 µl onto BHI agar (Cm10/Xgal100 µg/ml).

DAY 2.
Single colony purify 2x IMxxB + pIMAY-Z(insert).

DAY 3.
Screen for insert by colony PCR with MCS primers.
Inoculate a positive colony into 25 ml LB (+Cm10) and grow overnight at 37°C.
Inoculate a 10ml BHI broth with the target *S. aureus* strain and grow at 37°C.

DAY 4.
Miniprep pIMAY-Z(insert) and concentrate.
Make electrocompetent *S. aureus.*
Electroporate *S. aureus* with pIMAY-Z. Plate at 30°C for 48h.

DAY 6.
Pick two colonies from the 30°C plate, colony PCR screen with MCS primers and single colony purify.

DAY 8.
Pick a single colony, homogenise in 200µl of PBS and diluted to 10(-4). A 100µl aliquot of the undiluted was spread plated across one BHI agar (Cm10/Xgal100 µg/ml) and 10µl of the dilutions was spotted onto a second plate. Incubated overnight at 37°C.

DAY 9.
Pick six blue colonies from the 37°C integration plate and single colony purify.

DAY 10.
Screen the side of integration with AF/IM3 or DR/IM4. Pick one colony from either side of integration to inoculate 10ml BHI with blue colony and grow overnight at 30°C 200rpm.

Day 11.
Dilute the 10ml BHI and plate 2x10(-5) and 1x10(-6) on BHI agar (X-gal). Incubate overnight at 37°C.

Day 12.
Screen by colony PCR for the mutation with AF and DR primers on yellow colonies.
Single colony purify putative mutants.

DAY 13.
Inoculate BHI broth. Isolate genomic DNA. Confirm mutation by PCR. Send for WGS.

Supplementary Figure 2. PCR amplification of pIMAY-Z

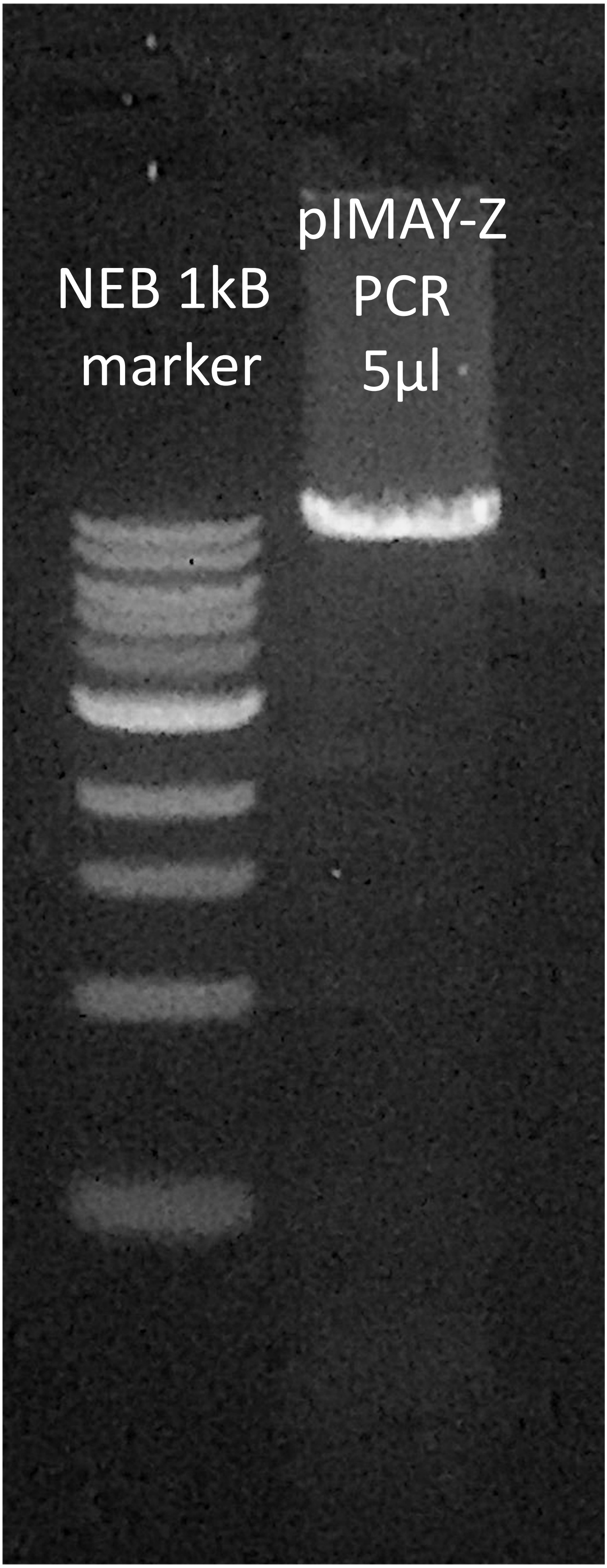

Supplement: Supplementary material 1 [file acmi-3-193-s001.pdf]
